# Contribution of S100A4-expressing fibroblasts to anti-SSA/Ro-associated atrioventricular nodal calcification and soluble S100A4 as a biomarker of clinical severity

**DOI:** 10.3389/fimmu.2023.1114808

**Published:** 2023-04-06

**Authors:** Christina E. M. Firl, Marc Halushka, Nicola Fraser, Mala Masson, Bettina F. Cuneo, Amit Saxena, Robert Clancy, Jill Buyon

**Affiliations:** ^1^ Division of Rheumatology, Department of Medicine, New York University Grossman School of Medicine, New York, NY, United States; ^2^ Department of Pathology, Johns Hopkins Medical Institutions, Baltimore, MD, United States; ^3^ Department of Obstetrics and Gynecology, University of Colorado Anschultz Medical Campus, Aurora, CO, United States

**Keywords:** congenital heart block (CHB), congenital heart block and neonatal lupus risk, cardiac fibroblast (CF), macrophage-fibroblast co-cultures, fibrosis, cardiac-NL, dystrophic calcification

## Abstract

**Background:**

Fibrosis and dystrophic calcification disrupting conduction tissue architecture are histopathological lesions characterizing cardiac manifestations of neonatal lupus (cardiac-NL) associated with maternal anti-SSA/Ro antibodies.

**Objectives:**

Increased appreciation of heterogeneity in fibroblasts encourages re-examination of existing models with the consideration of multiple fibroblast subtypes (and their unique functional differences) in mind. This study addressed fibroblast heterogeneity by examining expression of α-Smooth Muscle Actin (myofibroblasts) and of S100 Calcium-Binding Protein A4 (S100A4).

**Methods:**

Using a previously established model of rheumatic scarring/fibrosis *in vitro*, supported by the evaluation of cord blood from cardiac-NL neonates and their healthy (anti-SSA/Ro-exposed) counterparts, and autopsy tissue from fetuses dying with cardiac-NL, the current study was initiated to more clearly define and distinguish the S100A4-positive fibroblast in the fetal cardiac environment.

**Results:**

S100A4 immunostaining was observed in 4 cardiac-NL hearts with positional identity in the conduction system at regions of dystrophic calcification but not fibrotic zones, the latter containing only myofibroblasts. *In vitro*, fibroblasts cultured with supernatants of macrophages transfected with hY3 (noncoding ssRNA) differentiated into myofibroblasts or S100A4^+^ fibroblasts. Myofibroblasts expressed collagen while S100A4^+^ fibroblasts expressed pro-angiogenic cytokines and proteases that degrade collagen. Cord blood levels of S100A4 in anti-SSA/Ro-exposed neonates tracked disease severity and, in discordant twins, distinguished affected from unaffected.

**Conclusions:**

These findings position the S100A4^+^ fibroblast alongside the canonical myofibroblast in the pathogenesis of cardiac-NL. Neonatal S100A4 levels support a novel biomarker of poor prognosis.

## Introduction

Linking inflammation to fibrosis and/or calcinosis, fated end-stage sequelae in many autoimmune and immune-mediated rheumatic diseases, remains a challenge at the molecular level. Indeed, fibrotic replacement of the atrioventricular (AV) node and endocardial fibroelastosis in the late second trimester of pregnancy are histopathological lesions characterizing cardiac manifestations of neonatal lupus (cardiac-NL), also referred to as congenital heart block (CHB), associated with the transplacental passage of maternal anti-SSA/Ro antibodies (1). Molecular cascades to injury have been proposed and described as a complex interplay initiated by anti-SSA/Ro antibody opsonization of cardiac myocytes undergoing apoptotic remodeling and/or direct toxicity of cardiocytes by cross-reactivity with calcium channels (2–4). Opsonization crucially impairs physiologic autologous myocyte clearance of apoptotic cardiocytes (3), instead promoting macrophage phagocytosis and secretion of pro-inflammatory and pro-fibrotic mediators, stimulating transdifferentiation of nearby cardiac fibroblasts to myofibroblasts (2, 5–7). Myofibroblasts exhibit a scarring phenotype based on their expression of alpha-Smooth Muscle Actin (α-SMA) and contractile properties. What follows is a radical restructuring of the extracellular space, notably soft-tissue calcification and fibrosis irreversibly altering the conduction system of the fetal heart (1, 6). The kinetics of cardiac injury propagated by maternal anti-SSA/Ro antibodies is remarkably rapid, based at least on the clinical detection of a normal rhythm transitioning to incomplete block within 12 hours (8), and if progressed to complete block is ultimately immutable (9). In some cases of cardiac-NL, if not fatal, the disease progresses beyond birth, even after the complete disappearance of maternal antibodies from the infant’s circulation (10).

For the most part molecular investigation of the fibrotic paradigm in cardiac-NL has centered on the scarring myofibroblast (6, 7). However, by focusing solely on α-SMA-expressing fibroblasts, heterogeneity in the cardiac fibroblast population may be underappreciated. Although more extensively studied in the context of oncology and tumor migration (11) and less in autoimmune-associated scarring, another fibroblast-expressing protein, S100 Calcium -Binding Protein A4 (S100A4), merits consideration. S100A4 (also known as Fibroblast Specific Protein-1 and Metastasin-1) is a member of the S100 superfamily of calcium-binding proteins, generally known for their E-F hand motifs (11). S100A4 has no enzymatic activity but is social, forming dimers with itself and other family members, and with a variety of binding partners both intra and extracellularly (11, 12). S100A4’s intracellular targets include cytoskeletal elements (Non-muscle myosin II), adhesion contacts (Liprin β1), and the p53 nuclear phosphoprotein (11–13). Secreted extracellular S100A4, in its unique multimeric form, elicits the upregulation and secretion of proteolytic enzymes such as Collagenase-3 (MMP13), allowing for active remodeling of the extracellular matrix (ECM) to facilitate cell movement and vascular formation (14, 15). The protein is expressed and secreted by several cell types, macrophages and fibroblasts being of relevance to the pathogenesis of cardiac-NL. As a contributor to metastasis, S100A4 influences epithelial-mesenchymal transition as well as cell motility, invasion, and angiogenesis (15–17). In rheumatoid arthritis, an active feedback loop between inciting macrophages/leukocytes and synovial fibroblasts has been proposed in which S100A4 acts in an autocrine/paracrine manner to amplify matrix degradation and apoptosis, primarily through secretion of matrix metalloproteinases (MMPs) (18, 19). Moreover, S100A4 has been identified as a contributory factor in idiopathic pulmonary fibrosis wherein the protein is highly expressed in mesenchymal progenitor cells and crucial to their fibrogenic transition to idiopathic pulmonary fibroblasts (12, 20).

The current study was initiated to address the potential contribution of S100A4 to cardiac injury propagated by maternal anti-SSA/Ro antibodies. This was first approached by immunohistologic interrogation of 4 fetal hearts, from fetuses succumbing to cardiac-NL and a healthy heart electively terminated, for expression of the various fibroblast markers. An *in vitro* co-culturing system simulating a model of cardiac injury was leveraged wherein THP-1 macrophages were stimulated with an ssRNA— associated with SSA/Ro60 and a Toll-like receptor 7/8 agonist— and resulting supernatants incubated with human fetal cardiac fibroblasts (21, 22). Having established fibroblast S100A4 expression in addition to α-SMA in this model, further experiments were designed to dissect the various fibroblast phenotypes and functional consequences. Clinical translation of findings was assessed by the measurement of circulating S100A4 in neonates with cardiac-NL and those otherwise healthy, all exposed to maternal anti-SSA/Ro antibodies.

## Materials and methods

### Generation and transfection of human “macrophages”

The human monocytic cell line, THP-1, from the ATCC was cultured in RPMI 1640/10% FBS. THP-1 cells (4 x 10^5^/mL) were differentiated into a macrophage-like phenotype in 6-well plates with 0.2 µM phorbol-12-myristate-13-acetate (PMA) for 3 days followed by 48 hours in growth medium without PMA (23, 24). PMA-differentiated THP-1 cells were transfected with 2.5 µg ssRNA hY3 (a noncoding Y ssRNA binding Ro60 and Toll-like receptor 7/8 agonist), as described (22) for 18 hours. Transfection was carried out with a commercial kit (DOTAP Liposomal Transfection).

### Isolation, preparation, and treatment of fetal cardiac fibroblasts

Fibroblasts from healthy fetal human hearts (aged 16-24 weeks) were prepared as previously described (7). Using passages 2-5, fibroblasts were serum-starved for >2 hours then co-cultured at 1:5 dilution with the supernatants of PMA-differentiated THP-1 macrophages transfected with hY3 (“hY3 sup”) or with DOTAP alone (“NT sup”) for 72 hours in low serum (0.1% FBS) medium. Alternatively, fibroblasts were treated with activated Transforming Growth Factor-Beta 1 (TGF-β1) (2.7 ng/mL) (R & D Systems); IFNalpha (IFNα) (2000 U/mL) (PBL Science); a neutralizing antibody to IFNα (12 µM) (PBL Science, MMHA-2 clone); or recombinant S100A4 (0.5 µg/mL) (Abcam) added directly to the medium.

In immunofluorescence experiments, fibroblasts were initially plated (50,000/mL) on glass coverslips, serum-starved, treated, fixed with 4% paraformaldehyde and permeabilized with acetone. Coverslips were then blocked with 0.1% gelatin in PBS and stained with a Cy3-conjugated antibody to α-SMA (Sigma-Aldrich) (1:150) and a FITC-conjugated antibody to S100A4 (1:100) (BioLegend). DAPI (Sigma-Aldrich) was utilized to stain DNA content. Coverslips were mounted with Mowiol (Polysciences); images were captured using a ZEISS Axio Observer inverted light microscope at 20x magnification then processed with ImageJ software.

For flow cytometry assays, fibroblasts were plated (3 x 10^5^/mL) in 6-well plate(s), serum-starved, treated, permeabilized and stained using the same α-SMA and S100A4 conjugated antibodies as above. 10,000 events were acquired on an LSR II; cells were pretreated with Ghost Dye Violet 510 (Tonbo) to ensure cell viability. Data was analyzed with FlowJo software.

### Quantitative real-time PCR

RNA was isolated from human fetal cardiac fibroblasts (variously treated as detailed above but for 24 hours) using RNeasy Mini kit (Qiagen). 1 µg total RNA was used to prepare cDNA template with iScript cDNA Synthesis kit (BioRad). Primers for IL6, MMP13, IL10 and Col1α1 were selected based on primer sequences in the PrimerBank database (25). GAPDH was selected as an endogenous control. PCR reactions were performed in the StepOne Real-Time PCR System with Fast SYBR Green Master Mix (Thermo Scientific). The cycling conditions for all the genes were: 95°C for 3 min, 40 cycles of 95°C for 30 sec, 60°C for 20 sec, and 72°C for 30 sec. Relative changes in gene transcript were quantified as fold change based on 2^-ΔΔCt^ with relative expression of transcript normalized to GAPDH.

### Soluble collagen assay

Fibroblasts were spiked with collagen (12 µg/mL) as a positive control and to compensate for varying basal collagen secretion. After 30 hours of treatment with NT sup, hY3 sup, or recombinant S100A4, fibroblast supernatants were collected. The concentration of soluble collagen in the supernatants was measured using the Sircol soluble collagen assay (Biocolor). Absorbance was recorded with a multiwell plate reader set to 540 nm. The standard curve was obtained by running parallel 10, 25, 50 µg collagen standards.

### S100A4 feedforward signaling/conditioned media experiment

Untreated fibroblasts were plated on glass coverslips and treated for 12 hours with hY3 sup, NT sup or recombinant S100A4. The media was then aspirated and the cells were washed 3 times with PBS. After which, fresh growth media was added and the fibroblasts were cultured for 24 hours. The supernatants (conditioned media) of these were harvested and added to a new row of untreated fibroblasts on glass coverslips at 1:5 dilution. After another 24 hours these fibroblasts (co-cultured with the conditioned medium of the first round of fibroblasts) were fixed, permeabilized, stained, mounted, and photographed in accordance with the prior indirect immunofluorescence experiments.

### Evaluation of transcriptomic data sets

For evaluation of the transcriptome related to the different subtypes of fibroblasts, we examined a previously published data set (26) generated from human fetal fibroblasts plated at a density of 5 × 10^5^ cells/well (6-well plate), serum starved, and incubated for 16 hours with the following: no treatment, NT sup, hY3 sup, and TGF-β1. Similarly, we examined another previously published data set (26) wherein fibroblasts from cardiac-NL hearts were sorted using a cell-sorting flow cytometer yielding a DAPI-negative, CD45-negative, CD31-negative, and podoplanin-positive subset. Fold change (ratio of log_2_(transcripts per million)) was calculated relative to healthy hearts or untreated fibroblasts. For evaluation of the CHB leukocyte transcriptome related to vascular calcification, a previously published data set (27) generated from three healthy hearts and three CHB hearts—flow-sorted to yield a DAPI-negative, CD45-positive, podoplanin-negative, and CD31-negative population—was consulted.

### Tissue sections from fetal hearts

Formalin-fixed paraffin sections (6 μm section) were obtained from all hearts. Sections of right ventricle, left ventricle, and AV groove/conduction (septal) tissue were obtained from 4 fetuses dying with CHB (ranging from 20-27 weeks gestation) and a normal human fetus (no known cardiac disease) electively terminated at 24 weeks of gestation. Isotype control staining was conducted in sections from CHB Heart #2. Spleen tissue was used as a positive control for α-SMA and S100A4 immunostaining.

### Immunostaining

Deparaffinization was achieved by warming up paraffin sections for 1 hour at 60°C. Sections were sequentially treated with xylene and ethanol (30 min each). Endogenous peroxidase was inactivated by treatment with methanol + 1% H_2_O_2_ for 30 min. Sections were washed with PBS then epitopes were unmasked by hyaluronidase digestion (type IV-S, Sigma-Aldrich 1 mg/ml; 0.1 M Tris, pH 6.0) for 30 min. Slides were once again washed with PBS and incubated overnight at 4°C with primary Ab (anti-α-SMA, anti-S100A4, rabbit IgG isotype control) then incubated with alkaline phosphatase-conjugated anti-rabbit IgG (1 hour at room temperature). Sites of immunoreactivity were visualized using Fast Red TR/Naphthol AS-MX Tablet (Sigma-Aldrich) to report. The sections were counterstained with hematoxylin before photomicroscopy. Subsequent slides were stained with hematoxylin and eoisin or Masson’s Trichrome stain.

### S100A4 in cord bloods and supernatants *via* ELISA

Supernatants from THP-1 macrophages transfected with hY3 (hY3 sup) or DOTAP alone (NT sup) were assayed for secreted S100A4 *via* an in-house sandwich ELISA (blocked with 3% NFDM in PBS, developed with alkaline phosphatase and read with a multiwell plate reader at 405 nm). Serial dilutions of recombinant S100A4 (starting at 50 ng/mL) were run in parallel to generate a standard curve. This ELISA protocol was later expanded to umbilical cord blood samples (run in triplicate at 1:8 dilution) from (n=49) cardiac-NL and (n=47) healthy (anti-SSA/Ro-exposed) neonates. 1:8 dilution was determined after assay optimization. S100A4 ELISAs were run blinded to cord blood subject identification/respective severity score. Severity scores of clinical disease in the affected neonates were evaluated using a previously established severity score index that prioritizes most highly extranodal manifestations of cardiac-NL (endocardial fibroelastosis, dilated cardiomyopathy, and hydrops fetalis) with or without advanced AV block. Classification was based on disease status at birth. One neonate with transient mild EFE exposed to dexamethasone with a normal EKG was included in the no disease group. Cord bloods from three pairs of twin siblings with discordant disease were evaluated by ELISA; these pairs were color-coordinated on the graph accordingly. Data points were formatted to reflect medications taken by the mothers during affected and unaffected pregnancies, as follows: dexamethasone/betamethasone, hydroxychloroquine, dexamethasone/hydroxychloroquine in combination, dexamethasone/terbutaline in combination, IVIG, and dexamethasone/IVIG in combination. Neonates were categorized if exposed to maternal medication for any duration of the pregnancy. Many of the healthy neonates were part of the PATCH (Preventative Approach to Congenital Heart Block with Hydroxychloroquine) trial (28).

### sFRP-1 in cord bloods *via* ELISA

A selection of the same umbilical cord bloods as above was assayed for soluble Frizzled-Related Protein 1 (FRP-1) *via* sandwich ELISA commercial kit (Invitrogen). Bloods were diluted 1:6, run in triplicate, developed with horseradish peroxidase, and read with a multiwell reader at 450 nm in accordance with kit instructions. The same three pairs of twin siblings with discordant disease were evaluated for sFRP-1; the severity scores of clinical disease in the affected neonates were evaluated using the same previously established severity score index as above.

### Statistical analysis

Statistical significance was established *via* Kruskal-Wallis test for flow cytometry experiments; Mann-Whitney U test was applied for qPCR, soluble collagen assays, and ELISA experiments. Analyses were performed using GraphPad Prism version 10.7.2. Values of p <0.05 were considered significant.

## Results

### Histopathological features of end-stage cardiac-NL include novel S100A4^+^ fibroblasts at regions of dystrophic calcification

**Figure 1:**
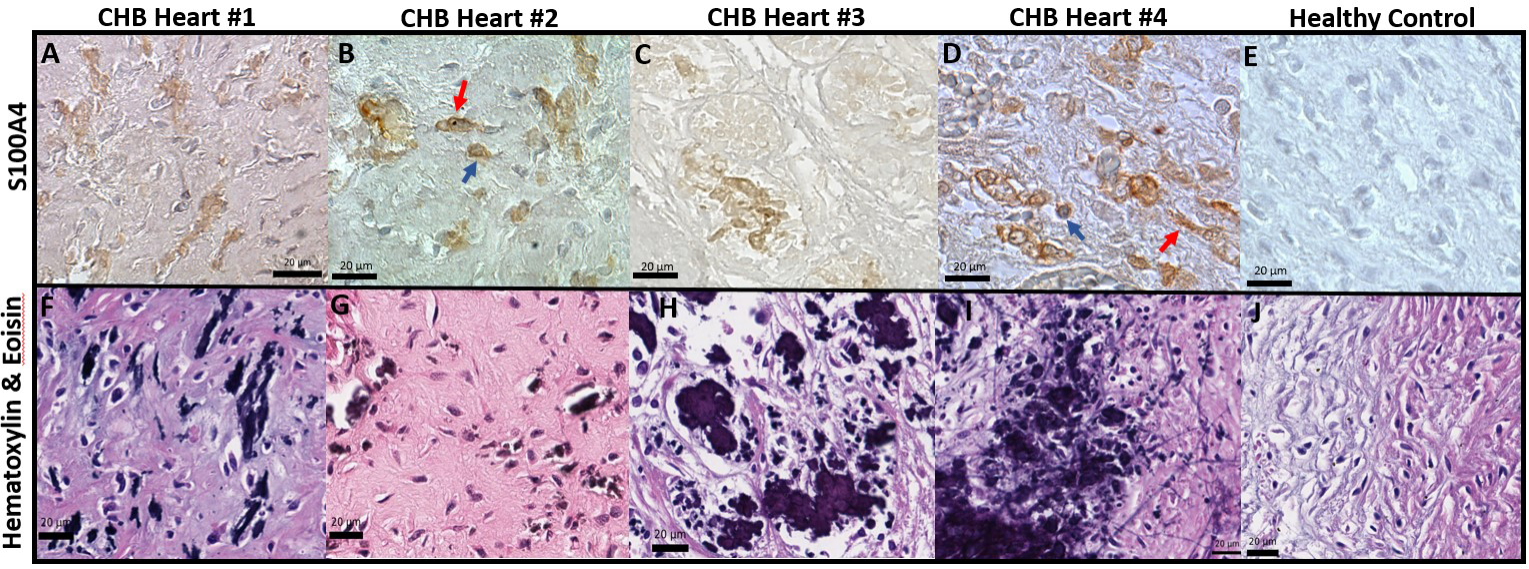
Histological features of end-stage cardiac-NL include S100A4^+^ macrophages and fibroblasts at areas with dystrophic calcification in the injured hearts. Human fetal hearts of offspring of 4 women enrolled in the RRNL were evaluated for pathology findings. Panels **(A-E)** are stains of AV nodal tissue from CHB Heart 1, CHB Heart 2,CHB Heart 3, CHB Heart 4 (**Table 1**), and an age-matched healthy control heart respectively. H&E staining of fetal heart tissue of the CHB cases **(F-I)** revealed properties in common include disruption to AV node and conduction tissue architecture associated with foci of dystrophic calcification. As expected, H&E staining of the healthy control heart **(J)** depicted normal nodal tissue unaffected by calcification. S100A4^+^ immunostains were evident at dense packs of cells (brown cells, **A-D**) surrounding microcalcification foci **(F-I)**. S100A4^+^ macrophage (plump sphere, blue arrow) and fibroblast (spindle-shaped, red arrow) identity was assigned on the basis of cell morphology **(B, D)**.

**Figure 2:**
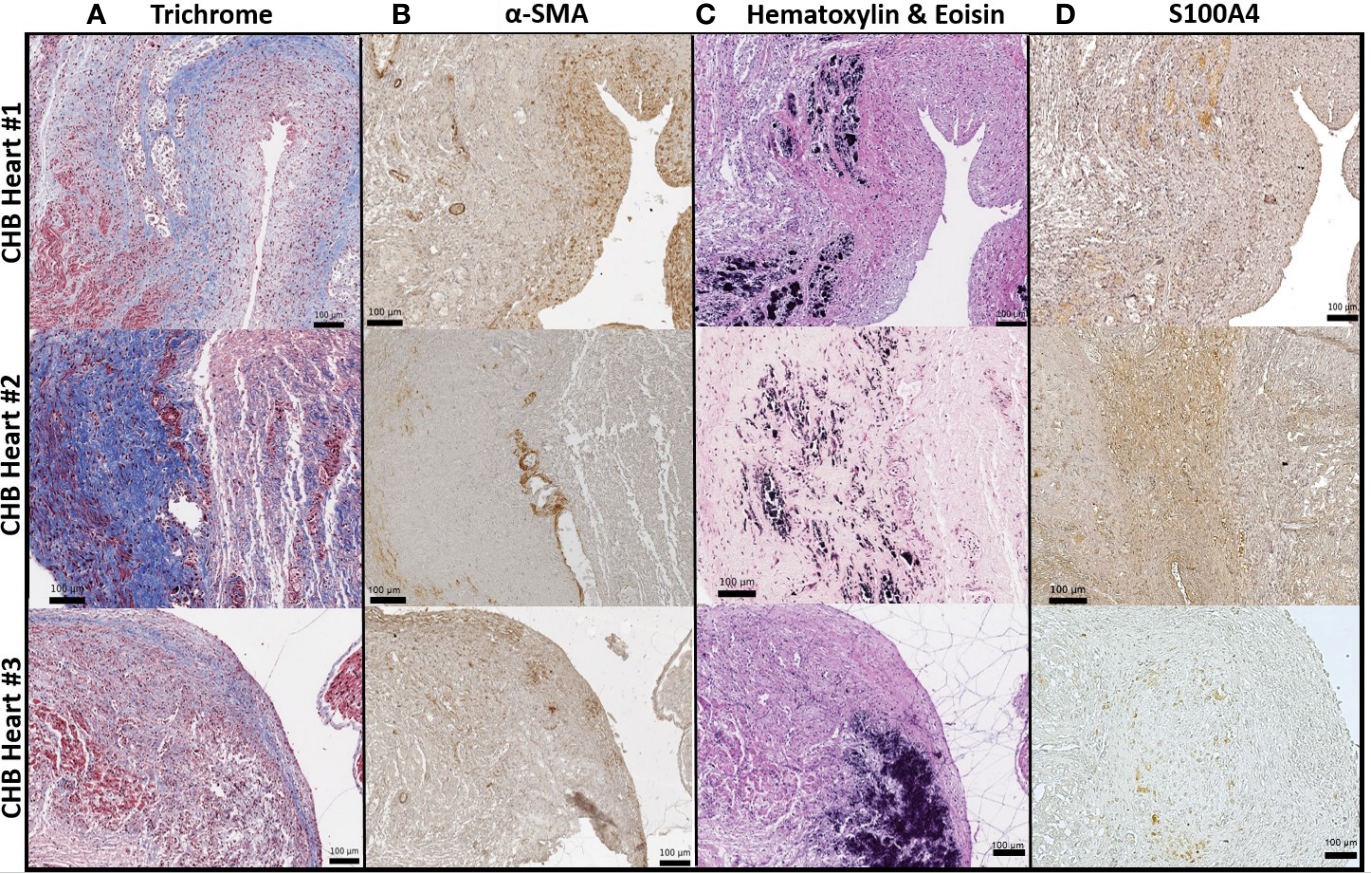
α-SMA^+^ fibroblasts and S100A4^+^ fibroblasts found largely at architecturally distinct sites of the injured heart. As in **Figure 1**, S100A4 positive expression **(D)** in the injured hearts was limited to regions with dystrophic calcification **(C)**, visualized by staining with hematoxylin and eosin. In contrast, α-SMA^+^ cells were localized to the epicardium (diffuse brown cells, **(B)** in zones with evident fibrosis, confirmed *via* Trichrome (Masson) staining **(A)**.

Previously reported fibrosis and calcification of the AV node on histologic evaluation of fetal hearts dying with cardiac-NL (1) motivated the interrogation of 4 similarly affected hearts (clinical features described in **Table 1**). Staining of AV nodal tissue with anti-S100A4 antibodies revealed expression in clusters of cells (**Figures 1A–D**) present only in areas with dystrophic calcification (**Figures 1F–I**) (calcification being a property in common among the cases). Cell morphology suggests S100A4 expression in both fibroblasts (spindle-shaped, red arrow) and macrophages (plump/sphere, blue arrow) (**Figures 1B, D**). Staining of AV nodal tissue of a matched healthy heart did not reveal S100A4^+^ cells (**Figure 1E**) or calcification (**Figure 1J**). Control for antibody isotype was conducted, using CHB Heart #2, to ensure S100A4 staining specificity (**Supplementary Figure 1**). Staining a section of membranous interventricular septum (CHB Heart #1) or AV node (CHB Hearts #2,3) with hematoxylin and eoisin (**Figure 2C**) and with Trichrome (Masson) (**Figure 2A**) presented a complex pattern of dystrophic calcification and collagen deposition respectively at distinct areas of the tissue, including portions of epi and endocardium. Staining with anti-α-SMA antibodies revealed diffuse expression (**Figure 2B**) consistent with the localization of classically-defined myofibroblasts in regions of fibrosis (the blue staining of Trichrome (Masson) used to identify collagen deposition). In contrast, staining of tissue with anti-S100A4 antibodies (**Figure 2D**) revealed expression in clusters of cells limited to areas with calcification, as in **Figure 1**. These histologic findings support separate anatomic locations, albeit it is acknowledged that α-SMA and S100A4 expression are not absolutely mutually exclusive, particularly in fields where fibrosis and calcification foci coincide. The next set of experiments addressed the hypothesis that there is a bifurcation of fibroblast roles leading to an anatomical dichotomy. To test this hypothesis directly, subsequent studies leveraged an *in vitro* model of cardiac-NL to evaluate whether anti-SSA/Ro autoantibodies provoke injury *via* a pathway involving S100A4^+^ fibroblasts.

**Table 1:** Chart review of notable features of 4 cases that are featured in this study.

CHB case	GA CHB Dx (wks)	V rate DX (bpm)	Nadir A/V (bpm)	GA demise (wks)	Anti-SSA/Ro60, Ro52 Anti-SSB/La48	Extranodal findings	Treatment/day
1	23	70	144/55	27	Anti-Ro60 (+) Anti-Ro52 (+) Anti-La48 (+)	Carditis; hydrops	10 mg Pred (23-24 wks) 4 mg Dex (24-27 wks)
2	20	60	130/50	27	Anti-Ro60 (+) Anti-Ro52 (+) Anti-La48 (-)	DCM; hydrops	4 mg Dex (21-22 wks) 20 mg Terb (26-27 wks)
3	18	70	110/40	20	Anti-Ro60 (+) Anti-Ro52 (+) Anti-La48 (+)	Pericardial effusion; hydrops	4 mg Dex (18.4-20 wks)
4	22	52	124/52	23	Anti-Ro60 (+) Anti-Ro52 (+) Anti-La48 (+)	Pericardial effusion	No Treatment

wks, weeks; A, atrial; V, ventricular; dex, dexamethasone; CHB, complete atrioventricular block; DX, diagnosis; GA, gestational age; pred, prednisone; terb, terbutaline; DCM, dilated cardiomyopathy.

### Phenotype of stimulated human fetal fibroblasts using an *in vitro* model of cardiac-NL

**Figure 3:**
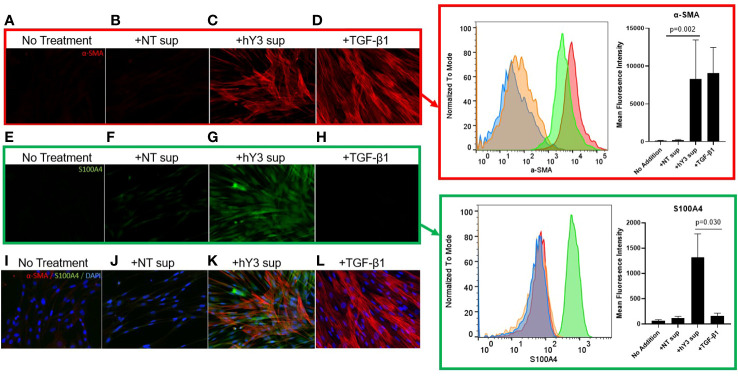
Differential effects of hY3-generated macrophage supernatants and TGF-β1 on fetal cardiac fibroblast expression of α-SMA and S100A4. Fibroblasts isolated from healthy fetal hearts were incubated with the supernatants of hY3-transfected macrophages (hY3 sup) for 72 hours or the direct addition of activated TGF-β1 and stained for α-SMA (red), S100A4 (green), and DAPI (blue), 20x magnification. Minimal staining for either α-SMA or S100A4 was observed in untreated fibroblasts **(A, E, I)** or with the addition of supernatants from vehicle alone (DOTAP) macrophages **(B, F, J)**. Incubation with hY3 sup **(C, G, K)** results in dual staining. Incubation with TGF-β1 results only in α-SMA expression **(D, H, L)**. Inset panels (right) show an authentication of immunofluorescence staining with flow cytometry. Flow panel (red box) shows untreated fibroblasts (blue histogram), like those incubated with NT sup (orange histogram), express negligible α-SMA. In contrast, fibroblasts cultured with hY3 sup (green histogram) or TGF-β1 (red histogram) exhibited a three-log-fold positive shift in α-SMA expression after 72 hours. Flow panel (green box) shows that exposure to hY3 sup resulted in the fibroblast expression of S100A4 (green histogram) while none of treatments including TGF-β1, shared this property. Quantification (mean fluorescence intensity) of each condition is shown in the bar graphs within the red and green boxes and represents the average of each condition over four flow cytometry experiments. Bars represent mean ± SEM.

Given macrophages and giant cells are frequently identified at calcified foci, our prior studies have incorporated macrophage activation and fibrosis as an *in vitro* model of cardiac-NL: ssRNA hY3 associated with SSA/Ro60 was leveraged as a proxy for immune complex uptake by macrophages and the addition of supernatants from these activated macrophages to cultured human fetal fibroblasts as a measure of macrophage-fibroblast crosstalk. When cultured with supernatants from hY3-transfected macrophages (hY3 sup), the fetal cardiac fibroblasts differentiated into two distinct populations, distinguished by the expression of α-SMA and/or S100A4 (**Figures 3C, G, K**). In contrast, direct addition of activated TGF-β1 to cardiac fibroblasts resulted in expression of α-SMA but not S100A4 (**Figures 3D, H, L**). Fibroblasts cultured in the absence of any additions (**Figures 3A, E, I**) or following treatment with supernatants of macrophages transfected with DOTAP alone (NT sup) did not generate substantial expression of either α-SMA or S100A4 (**Figures 3B, F, J**).

Flow cytometry was used to confirm the differential effects of the variously generated supernatants. As with the indirect immunofluorescence, fibroblasts co-cultured with hY3 sup exhibited marked expression of both α-SMA (red box, red histogram) and S100A4 (green box, green histogram) whereas fibroblasts treated with TGF-β1 only demonstrated a shift in the expression of α-SMA (**Figure 3**, red box, red histogram). No addition and NT sup fibroblasts revealed marginal expression of either marker (**Figure 3**, red, green boxes). The sizeable shift in α-SMA expression in both hY3 sup (Mean Fluorescence Intensity: 8263 ± 5185) and TGF-β1 (9085 ± 5867) conditions was quantified relative to baseline (117.0 ± 48.3) and NT sup (207.3 ± 62.5) conditions and found to be significant (**Figure 3**, red box, bar graph, NT sup vs hY3 sup, p=0.002, n=4). The significant shift in S100A4 expression unique to fibroblasts co-cultured with hY3 sup (1316 ± 469) was similarly quantified versus the other three conditions (**Figure 3**, green box, bar graph plot, hY3 sup vs TGF-β1, p=0.030, n=4). Neither NT sup (122.8 ± 30.6) nor TGF-β1 (159.2 ± 56.9) resulted in any significant increase in S100A4 expression relative to untreated fibroblasts (64.8 ± 22.2) (**Figure 3**, green box, bar graph plot, n=4).

**Figure 4:**
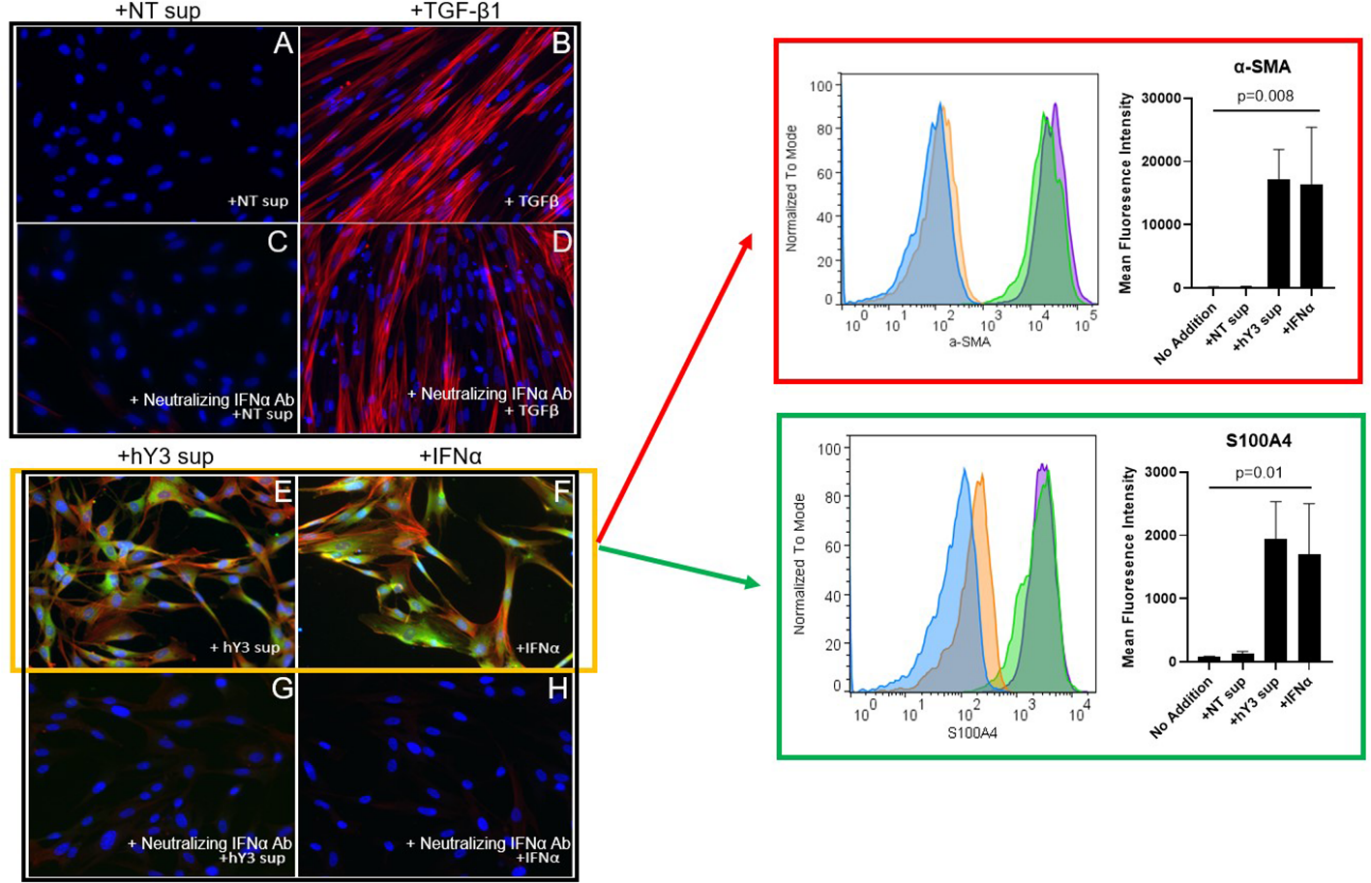
Phenotype of hY3-generated macrophage supernatants but not TGF-β1 on fetal cardiac fibroblasts is sensitive to co-incubation with a neutralizing anti-interferon antibody. Fibroblasts isolated from healthy fetal hearts were incubated with hY3 sup or IFNα for 72 hours and then stained for α-SMA and S100A4 by indirect immunofluorescence **(E, F)** or flow cytometry (red and green boxes). No expression for either α-SMA or S100A4 was observed with the addition of NT sup **(A)**. Incubation with supernatants from hY3-transfected macrophages again resulted in dual positive shifts in α-SMA and S100A4 expression **(E)**. The direct addition of IFNα, unlike TGF-β1 **(B)**, also generated dual expression of both markers **(F)**. Panels within red, green boxes show the quantification of the average (mean fluorescence intensity) over three flow cytometry experiments. Conditions at bar graphs represent mean ± SEM. In sum, the flow cytometry authenticated the results using immunofluorescence staining. A neutralizing antibody to IFNα was added to each of the four conditions **(C, D, G, H)**. The neutralizing antibody to IFNα prevented the dual generation of S100A4, α-SMA populations in fibroblasts treated with IFNα **(G)** as well as with hY3 sup **(H)**. TGF-β1-stimulated α-SMA expression was unaffected by the neutralizing antibody **(D)**.

Since previous scRNA-seq of fetal heart(s) dying with cardiac-NL identified increased expression of type I IFN-stimulated genes (ISGs) in several cell types, including fibroblasts, (26, 29), we added human IFNα directly to fetal cardiac fibroblasts and again stained for α-SMA and S100A4 *via* indirect immunofluorescence. In contrast to the direct addition of TGF-β1, IFNα induced dual differentiation of the fibroblasts, with expression of both α-SMA and S100A4, paralleling the results obtained with hY3 sup (**Figures 4E, F**). Again, TGF-β1 induced myofibroblast transdifferentiation only (**Figure 4B**). The significant mean expression of α-SMA and S100A4 in response to IFNα (α-SMA: 16332 ± 9085; S100A4: 1698 ± 806.4) and hY3 sup (α-SMA: 17101 ± 4824; S100A4: 1939 ± 594.6), as assayed via flow cytometry, were quantified (n=3) compared to untreated fibroblasts (α-SMA: 117.0 ± 48.3; S100A4: 64.8 ± 22.2) (**Figure 4**, α-SMA, red box, bar graph; S100A4, green box, bar graph). Moreover, dual differentiation staining after treatment with hY3 sup and IFNα was completely abolished with a neutralizing IFNα antibody (**Figures 4G, H**), supporting that type I IFN is released by macrophages downstream of the uptake of SSA/Ro immune complexes. In contrast, the α-SMA expression induced by treatment with TGF-β1 was unaffected by neutralizing antibodies to IFNα (**Figure 4D**).

### Transcriptomic and functional characterization of fetal fibroblasts expressing S100A4 and/or α-SMA

**Figure 5:**
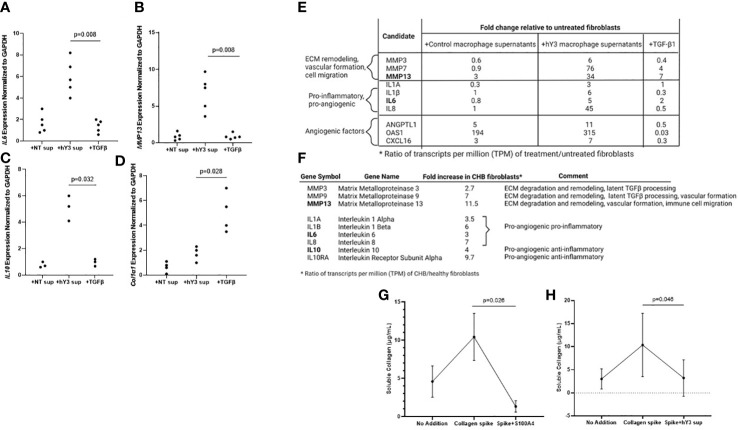
Fibroblasts stimulated with hY3 macrophage supernatants, similar to those flow-sorted from cardiac-NL hearts, display a matrix-remodeling phenotype based on transcriptomic and cell biological evaluations. Transcripts of IL6 **(A)**, MMP13 **(B)**, IL10 **(C)**, and COL1α1 **(D)** were measured in fibroblasts after varied treatment (24 hours) with macrophage supernatants (NT/DOTAP, 1:5 vol/vol), hY3-treated macrophage supernatants (1:5), activated TGF-β1 (2.7 ng/mL), or recombinant S100A4 (0.5 µg/mL). The y-axis represents 2^-ΔΔCt cycle threshold values of the target transcript normalized to GAPDH. p-values are indicated for comparisons of hY3 sup vs NT sup using Mann-Whitney U test. Transcripts IL6 and MMP13 (bold) are among other matrix-remodeling and pro-angiogenic transcripts increased in fibroblasts treated with hY3 macrophage supernatants relative to untreated fibroblasts **(E)** as assessed *via* bulk RNA sequencing. Bulk RNA sequencing on fibroblasts flow-sorted from CHB hearts similarly showed increased expression of matrix-remodeling MMPs and pro-angiogenic cytokines, including IL6, IL10, and MMP13 **(F)**. Panels **(G, H)** show functional evaluations: resting and stimulated fibroblasts were exposed to soluble collagen (12 µg/mL). After 30 hours, the supernatants of the variously treated fibroblasts were assayed for soluble collagen. Both fibroblasts treated with S100A4 **(G)** and fibroblasts cultured with hY3 macrophage supernatants **(H)** exhibited collagenase activity in their significant degradation of the collagen bolus. The bar graphs at Panels **(G, H)** represent mean ± SEM, over five experiments. The analysis of association of collagen levels and treatment of fibroblasts with exogenous S100A4 or supernatants of hY3-treated macrophages was analyzed by Mann-Whitney U test for n=5 experiments.

The next set of experiments addressed the cytokine expression profile of fibroblasts stimulated with hY3 sup or TGF-β1 directly, as agents that selectively induce S100A4^+^ dominant and α-SMA^+^ fibroblasts, respectively. After 24 hours in culture, fibroblasts exposed to hY3 sup significantly upregulated (mRNA) expression of the pro-angiogenic cytokine IL6 compared to treatment with TGF-β1 (5.7 vs. 1.5, p=0.0079, n=5) (**Figure 5A**). Likewise, expression of MMP13, a matrix metalloproteinase involved in ECM remodeling (crucial for subsequent neovascularization) and cell motility (14, 30, 31), was significantly increased after exposure to hY3 sup (hY3 sup vs. TGF-β1: 7.5 vs. 0.8, p=0.0079, n=5) (**Figure 5B**). Expression of IL10, an anti-inflammatory pro-angiogenic cytokine, was also upregulated in fibroblasts co-cultured with hY3 sup but not in fibroblasts treated with TGF-β1 (hY3 sup vs. TGF-β1: 5.1 vs. 0.96, p=0.032, n=3) (**Figure 5C**). In contrast, expression indicative of collagen/ECM deposition (Col1α1) was upregulated by TGF-β1 compared to hY3 sup (4.8 vs. 1.8, p=0.028, n=4) (**Figure 5D**). Notably, the qPCR data harmonized with bulk RNA sequencing conducted on fibroblasts treated with the same conditions (NT sup, hY3 sup, TGF-β1). Specifically, fibroblasts treated with hY3 sup uniquely upregulated ECM-degrading matrix metalloproteinases, including MMP13 (**Figure 5E**), and pro-inflammatory, pro-angiogenic cytokines, including IL6, as well as angiogenic factors previously reported in the S100A4^+^ cardiac fibroblast transcriptome (21). When flow-sorted cardiac fibroblasts from 3 CHB hearts and 3 healthy hearts were similarly sequenced, the same pattern of upregulated MMPs and pro-angiogenic cytokines was observed in CHB fibroblasts relative to the healthy control (**Figure 5F**). Transcripts common among qPCR and RNAseq experiments (MMP13, IL6, IL10) are bolded.

Given the upregulation of ECM-degrading transcripts in CHB fibroblasts and those exposed to hY3 sup, further phenotypic distinction between the fibroblast populations was generated using the SirCol soluble collagen assay. In considering that there might be functional differences between α-SMA^+^ fibroblasts that secrete collagen and S100A4^+^ fibroblasts, we tested whether the direct addition of recombinant human S100A4 to cardiac fibroblasts would minimize the level of soluble collagen in the cell medium. In order to compensate for variable basal production of collagen in cardiac fibroblasts, fibroblasts were treated with collagen (12 µg/mL) to ensure a positive control. Incubation with S100A4 for 30 hours resulted in marked collagen degradation (Collagen spike vs. S100A4+spike: 14.1 vs. 0.6 µg/mL, p=0.026, n=5) (**Figure 5G**). Fibroblasts treated with hY3 sup similarly exhibited collagenase activity, to a lesser degree (Collagen spike vs. hY3 sup+spike: 10.4 vs. 4.2 µg/mL, p=0.046, n=5) (**Figure 5H**).

### S100A4-induced cytokine secretion and feedforward behavior

**Figure 6:**
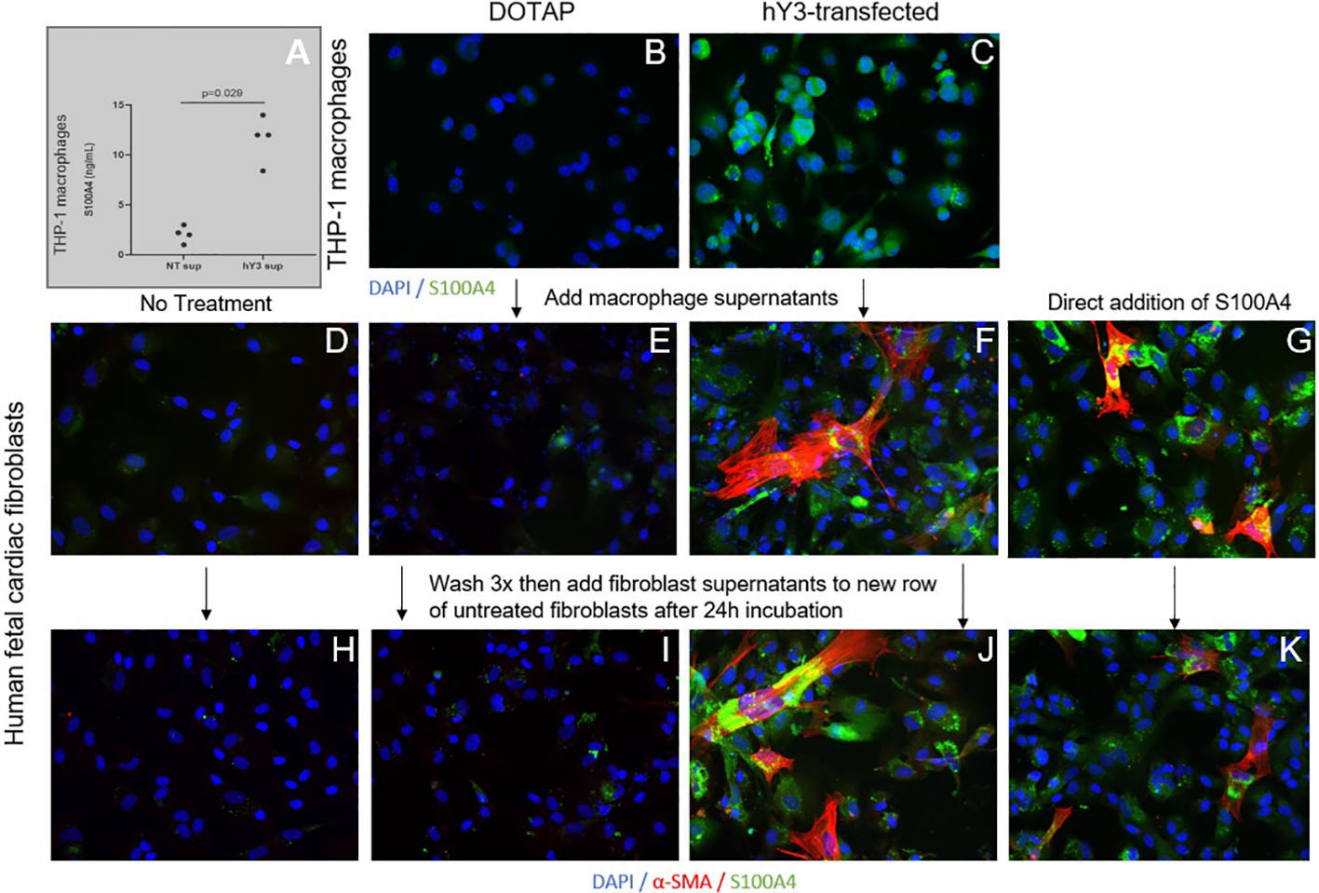
Influence of hY3-transfected macrophages and hY3 sup exposed-fibroblasts on surrounding cells. Evaluation of S100A4 in the supernatants of untreated macrophages (NT sup) and macrophages transfected with hY3 (hY3 sup) was performed using a specific enzyme-linked immunosorbent assay **(A)**. Levels of S100A4 were significantly greater in the supernatants of the hY3-transfected macrophages compared to untreated, a result that harmonized with immunofluorescence of resting and stimulated THP-1 macrophages wherein S100A4 expression was observed in hY3 treated-macrophages but not in the vehicle-treated condition (**B, C**, respectively). The ability of S100A4-secreting cells to influence neighboring fibroblasts was investigated *via* the generation of conditioned medium. Untreated fibroblasts **(D)** along with fibroblasts cultured with NT sup **(E)**, hY3 sup **(F)** or with recombinant S100A4 **(G)** were incubated for 12 hours then washed three times with PBS and incubated with fresh medium for 24 hours. After 24 hours the supernatants of each of the four fibroblast conditions were harvested and added to a fresh row of untreated fibroblasts, which were incubated for 24 hours and then stained for α-SMA (red), S100A4 (green), DAPI (blue) at 20x magnification. As shown in Panels **(F, J, G, K)**, fibroblasts with exposure to hY3 sup or S100A4 conditioned mediums respectively, thereby express S100A4 and secrete S100A4 into the medium **(J, K)**, influencing the phenotype of surrounding cells. Panels **(D, E, H, I)** display no "conditioning" related to basal fibroblasts and those treated with NT sup.

Given the distinct effects of hY3 sup, IFNα and TGF-β1 on fibroblast expression and behavior, the next set of experiments addressed the secretion of S100A4 by the stimulated macrophage supernatants. Evaluation of THP-1 supernatants for S100A4 *via* enzyme-linked immunosorbent assay highlighted the secreted protein’s presence in hY3-stimulated supernatants over DOTAP-transfected (13 vs. 3 ng/mL, n=4, p<0.01) (**Figure 6A**). Similarly, THP-1 macrophages treated with hY3, but not vehicle control, highly expressed S100A4 (**Figures 6B, C**). An indirect approach to evaluate S100A4 in fibroblast cultures involved a ‘two-tier’ double round culture condition. Briefly, at stage one, untreated cardiac fibroblasts were exposed to no addition, NT sup, hY3 sup, or recombinant S100A4 (added directly) (**Figures 6D–G**). At the second stage, untreated fibroblasts received the previous stage’s fibroblast supernatants; whereby conditioned media involving exposure to hY3 sup or recombinant S100A4, resulted in the untreated fibroblasts themselves expressing S100A4 and α-SMA, evidencing the feedforward amplification of the S100A4 signal *in vitro* (**Figures 6J, K**). No 'conditioning' related to basal fibroblasts and those treated with NT sup was observed (**Figures 6D, E, H, I**).

### S100A4 as a potential biomarker of cardiac-NL

**Figure 7:**
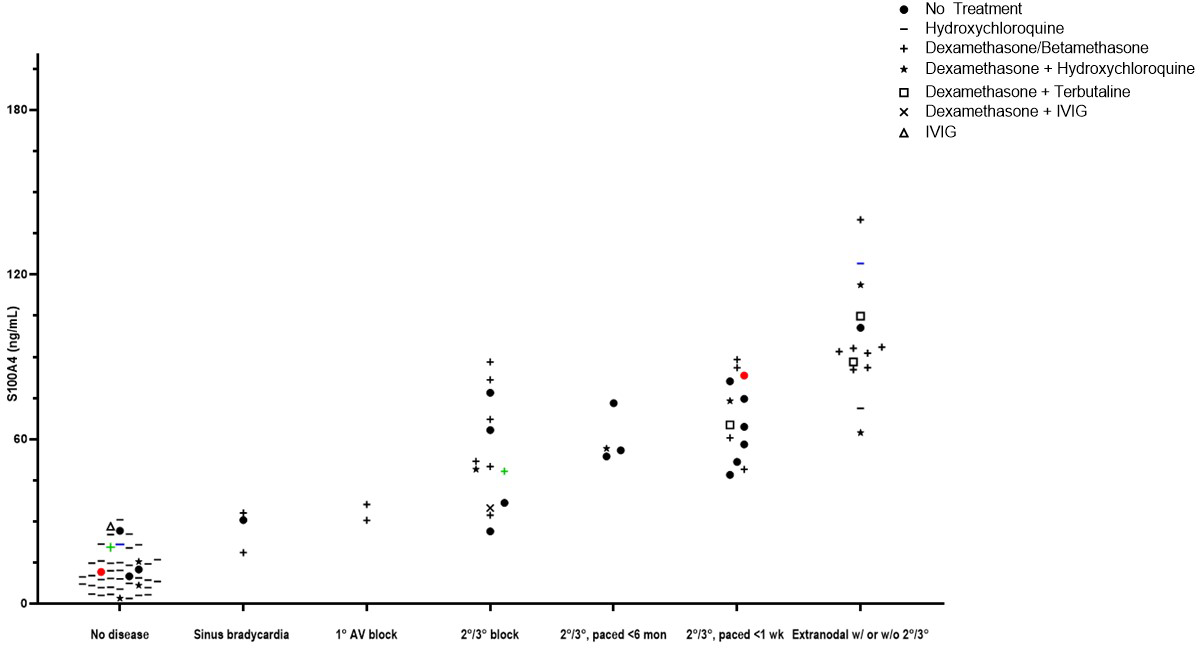
Evaluations of cord blood with an association of levels of S100A4 and severity of disease. Access to a cohort of anti-Ro+ mothers at delivery who consented to donate umbilical cord blood of their neonate was leveraged to interrogate levels of S100A4 and fetal manifestations. Each point represents an individual subject. A comparison of median levels of S100A4 detected in the (n=49) cardiac-NL neonates compared to the (n=47) anti-SSA/Ro+ exposed, unaffected neonates was conducted *via* the Mann-Whitney U test (61.6 ng/mL vs. 12.0 ng/mL respectively, p<0.0001). At the x-axis, groups of subjects appear from low to high severity. In addition, there are colored dots representing discordant twin pairs. Maternal treatments during pregnancy are reflected in the data points and defined in the legend insert on the graph.

Clinical translation of the newly-identified S100A4^+^ fibroblast phenotype was actualized with ELISA measurement of 96 umbilical cord samples from anti-SSA/Ro-exposed neonates (healthy, n=47 (49%); cardiac-NL, n=49 (51%)). Soluble S100A4 was significantly elevated in the cord bloods of neonates with cardiac-NL compared to those with no evidence of injury (61.6 ng/mL vs. 12.0 ng/mL respectively, p<0.0001) (**Figure 7**). Cord blood levels of S100A4 tracked with a previously established severity index of cardiac-NL disease (32). Specifically, 12 of 14 (86%) neonates with the most severe scores—advanced AV block and/or at least one extranodal manifestation—have the highest serum levels of S100A4 (>86 ng/mL); the lowest of the 14 being a milder case (endocardial fibroelastosis with no block) (**Figure 7**). Remarkably, for 3 twin pairs (all with discordant disease), the level of S100A4 was consistently higher in the affected twin compared to the unaffected twin. Treatments given to the mothers of affected and unaffected neonates at any time during the pregnancy are reflected in the data symbols. For the affected neonates, the most common treatment was dexamethasone, given to 31 of 49 (63%) mothers. There was no significant relationship between cumulative dose of dexamethasone and the level of S100A4 (**Supplementary Figure 2**). Another secreted protein, Frizzled-like Receptor Protein-1 (sFRP-1), which acts as an antagonist of the Wnt/β-catenin signaling pathway, was evaluated with the expectation that it would track with severity (33, 34). In contrast to S100A4, sFRP-1 showed no association with degree of injury and the levels were equal to those seen in the healthy neonates (**Supplementary Figure 3**).

## Discussion

Increased appreciation of the heterogeneity in fibroblasts (35–39) calls for re-examination of existing pathological cascades with consideration of multiple fibroblast subtypes and their functional differences. To date the focus on fibroblasts in anti-SSA/Ro-mediated cardiac scarring has been largely dominated by the α-SMA^+^ myofibroblast (6, 7). However, evaluation of 4 fetal hearts dying with cardiac-NL revealed two distinct fibroblast phenotypes with α-SMA expression dominant in fibrotic zones and S100A4 primarily detected in regions with dystrophic calcification. Exploiting an *in vitro* model of cardiac-NL—supernatants from macrophages stimulated with the ssRNA hY3, associated with SSA/Ro60 as a proxy for immune complex uptake (21, 22)—promoted two distinct fibroblast populations, characterized by the expression of α-SMA and/or S100A4. This dual differentiation signature proved unique to the SSA/Ro-informed macrophage-fibroblast dialogue as fetal cardiac fibroblasts treated with TGF-β1 alone generated only α-SMA^+^ myofibroblasts. That direct addition of IFNα, similar to the effect of hY3 macrophage supernatants, generated both fibroblast populations reinforces previous work positioning type I Interferon in this cellular exchange (26, 29). Neutralizing interferon attenuated the activation phenotype of both fibroblast subtypes induced by hY3 sup, a result suggesting that prior to a bifurcation of fibroblasts, there is feedforward signaling by IFN, which contributes to regulating inflammatory and pro-fibrosing milieu in cardiac-NL. This result also suggests that type I IFN is the dominant cytokine released in hY3 supernatants. Moreover, neonatal S100A4 levels provided a novel biomarker of poor prognosis.

Even among as diverse a cell type as fibroblasts, cardiac fibroblasts are notoriously plastic in phenotype (39–45). The extracellular matrix (ECM), long bracketed as a uniform interstitial tapestry, continues to evince itself as a sensitive, dynamic provider of the crucial context for proper cardiac function—intractably responsive to the cardiac fibroblast and vice versa—bringing heightened attention to the chief architects of the stromal space (39–45). Compelling work to delineate reported cardiac fibroblast subtypes and their appearance post-injury has been done in the context of myocardial infarction (MI) (17, 39, 41, 44, 45). Specifically, Saraswati et al. demonstrated two distinct cardiac fibroblast populations with varying transcriptional predispositions and cellular origins post-MI: S100A-expressing fibroblasts and the canonical α-SMA^+^ scarring myofibroblasts (17). In contrast to the latter’s prioritization of matrix synthesis/collagen deposition, S100A4^+^ cardiac fibroblasts upregulated targets involved in cell-cell adhesion/migration, ECM degradation and remodeling, particularly MMP9 and MMP13 (17). The secretion of MMPs into the extracellular space and resultant degradation of the ECM is a crucial first step in chemotaxis, immune cell migration, neovascularization, and even processing of latent TGFβ (14). This subset of fibroblasts additionally upregulated angiogenesis modulators IL6 and IL10. A more in-depth molecular understanding of cardiac-NL may similarly require considering fibroblast function beyond simply laying down of scar tissue.

To approach the roles of potential fibroblast subtypes in cardiac-NL, we leveraged Saraswati et al.’s studies to choose candidates for our own quantitative real-time PCR experiments. Having observed two distinct populations in immunofluorescence experiments, the marked upregulation of IL6 and IL10 in fibroblasts cultured with hY3 sup confirmed the presence of pro-angiogenic S100A4^+^ fibroblasts downstream of the initial anti-SSA/Ro autoantibody insult. Consistent with our qPCR results, bulk RNA sequencing of fibroblasts cultured with hY3 sup and those flow-sorted from 3 hearts with CHB revealed increased expression of IL6, IL10, and MMP13 plus additional candidates implicated in the S100A4+ transcriptome (MMP3, MMP9, IL10Rα, IL-1β, IL-1α, IL8, ANGPTL1, CXCL16) (17) compared to untreated cardiac fibroblasts or fibroblasts sorted from 3 healthy hearts respectively.

Conditions in which S100A4-expressing fibroblasts are generated (hY3 sup or recombinant, S100A4 added directly to fibroblasts) exhibited degradation of a bolus of soluble collagen, leveraging the transcriptional upregulation of MMPs, like MMP13, into tangible proteolytic maneuvering. It is worth noting that the collagenase activity of fibroblasts treated with recombinant S100A4 “outpaced” that of fibroblasts co-cultured with hY3 sup. Perhaps this is a feature of the playing-out of dueling phenotypes (S100A4 vs α-SMA) that arise downstream of the initial anti-SSA/Ro autoantibody insult. At the same time, the early predominance of an ECM-degrading fibroblast subtype is in keeping with the temporal sequence of fibroblast response post-MI, generally depicted as follows: pro-inflammatory, pro-angiogenic→myofibroblast→anti-inflammatory→return to homeostasis (39, 41). Likewise, Saraswati et al. reported S100A4^+^ fibroblasts appeared first and predominated over myofibroblasts within the first 72 hours of injury. Given we assayed for soluble collagen after 30 hours of incubation with hY3 sup, this may explain why hY3 sup-exposed fibroblasts more closely resembled direct addition of S100A4. In contrast, our immunofluorescence images were captured after 72 hours, perhaps explaining the emergence of the myofibroblast.

With no robust mouse model of cardiac-NL as yet established in which histology of an anti-SSA/Ro pup heart exhibits fibrosis, we mined human fetal autopsy tissue to demonstrate S100A4 expression. Locating S100A4 in 4 second-trimester hearts of fetuses that succumbed to cardiac-NL—but not in a healthy heart—again pointed to S100A4 expression as a post-injury response. Furthermore, the expression of S100A4 in cardiac conduction tissue (i.e. AV node and conduction axis) and surrounding areas (i.e. atrial wall and interventricular septum) reinforces S100A4’s unique relevance to maternal anti-SSA/Ro-propagated cardiac injury. Moreover, S100A4 and α-SMA’s generally distinct anatomic localizations and the differing pathological architecture associated with each (calcinosis versus fibrosis respectively), supports the idea that the two populations have distinct pathogenic roles. Given S100A4 expression colocalized with areas of dystrophic calcification in the autopsy tissues, it may be that calcification is the pathogenic outcome of defective angiogenesis. Although the molecular origins of soft-tissue calcification remain unclear in homeostatic tissue, defective angiogenesis has long been implicated in the pathological cascade (45). The pro-angiogenic cytokine program of the S100A4^+^ fibroblast includes IL6, which has been shown to be as influential an angiogenic factor as VEGF (46–50), and has been tied to insufficient pericyte coverage in developing vasculature (45). Additionally, bulk RNA sequencing of flow-sorted leukocytes from 3 CHB hearts revealed overexpression of BMP signaling transcripts associated with vascular calcification (**Supplementary Figure 4A**) (51, 52). An agnostic bioinformatics inspection of the top 500 transcripts ranked by fold change of expression in CHB leukocytes revealed the enrichment of five gene ontology (GO) categories of relevance to our theory of S100A4-driven pathogenesis including extracellular matrix, collagen catabolic process, metalloendopeptidase (MMP) activity, cellular response to BMP stimulus and BMP signaling (**Supplementary Figure 4B**). Though it may seem counterintuitive that an ECM-degrading fibroblast is implicated in fibrotic injury, S100A4 is similarly overexpressed in lung tissue of idiopathic pulmonary fibrosis patients as well as in cardiac hypertrophy and kidney fibrosis (53–55). However, it may also be, as we posit, that calcification and fibrosis represent differing pathological cascades commonly downstream of the initiating autoantibody insult.

Further *in vivo* relevance of the S100A4-expressing fibroblast in the pathogenesis of cardiac-NL was obtained by evaluation of cord blood from both affected and unaffected neonates (inclusive of twins discordant for disease) exposed to maternal anti-SSA/Ro. The significant elevation of S100A4 in the cord bloods of cardiac-NL compared to unaffected neonates and the tracking of S100A4 levels with severity of cardiac injury may reflect the multiple sources of S100A4 secretion (e.g. macrophages and fibroblasts) in the pathogenesis of cardiac-NL and sustained irreversible injury loop whereby S100A4 self-perpetuates in an autocrine/paracrine amplification manner. This may explain the lower levels observed in our *in vitro* macrophage cultures compared to the cord bloods. Precedent for the clinical application of secreted S100A4, this protein has also been reported to be elevated in the bronchoalveolar fluid of patients with idiopathic pulmonary fibrosis and in the blood of patients with rheumatoid arthritis (18, 53). Elevated levels of S100A4 have also been demonstrated in patients with chronic hepatic fibrosis (56) albeit lower than in the cardiac-NL cord bloods.

Several limitations of our study need to be acknowledged. The cardiac fibroblasts are isolated from hearts of healthy abortuses. These fibroblasts, used in our *in vitro* studies, generate a double-positive (S100A4^+^ α-SMA^+^) population in addition to the single-positive subtypes we focus on in this manuscript. It is for this reason that we referred to S100A4 and α-SMA fibroblast expression resulting from treatment with hY3 sup or IFNα as “and/or”. This double-positive population, whose relevance *in vivo* remains to be further delineated, may represent an influence of *in vitro* methods—plating fibroblasts on plastic rather than evaluation in hydrogel— or support a chronology to fibroblast subtype generation, similar to that in MI as we outlined above. Affected hearts are from fetuses dying *in utero* (a rare event) and even hearts from otherwise electively-terminated healthy fetuses are prioritized to standard-of-care autopsy evaluations or totally unobtainable and thus are particularly challenging to procure. We did not have access to cord bloods from anti-SSA/Ro-negative neonates. Although the purpose of our evaluation was to identify whether S100A4 is a potential biomarker of injury and prognosis, it may be that even the unaffected anti-SSA/Ro^+^ neonates have a very low but clinically insignificant level of secreted S100A4. This is possible since IFN signaling in fetal macrophages has been noted in healthy anti-SSA/Ro -exposed neonates (57).

In sum, based on novel data presented herein and our previous studies, we propose the following scenario linking anti-SSA/Ro antibodies to cardiac injury (**Supplemental Figure 5**). Opsonization of cardiac myocytes impairs autologous cardiomyocyte clearance which attracts macrophages and resultant secretion of pro-fibrotic and pro-inflammatory cytokines driving nearby cardiac fibroblasts to dually differentiate into α-SMA and S100A4-expressing populations. While the former initiates deposition of collagen and other ECM proteins, the latter’s generation of proteolytic effectors, namely MMPs, results in a dense microenvironment of ECM-degrading activity paving the way for chemotaxis/immune cell migration and angiogenesis. The pro-angiogenic and distinct collagenase activity may represent a thwarted attempt to early on mitigate fibrotic sequelae and/or hypoxia induced by the collagen-secreting myofibroblast with resultant unintended soft-tissue calcification. Alternatively, evolving literature concerning the pro-fibrotic activity of MMPs, despite their canonical role in ECM degradation, such as facilitating immune cell migration, cytokine signaling, and activating latent TGFβ secreted into the extracellular space, supports S100A4^+^ fibroblast's role in furthering cardiac fibrotic injury (58). We speculate that the S100A4-expressing fibroblast population stands to explain various dueling signatures previously unexplained by myofibroblast homogeneity. Moreover, neonatal S100A4 levels support a novel biomarker of poor prognosis.

## Clinical perspective

Neonatal S100A4 levels support a novel biomarker of poor prognosis for anti-SSA/Ro -associated cardiac-NL.

## Data availability statement

The original contributions presented in the study are included in the article/**Supplementary Material**. Further inquiries can be directed to the corresponding author.

## Ethics statement

The studies involving human participants were reviewed and approved by NYU Institutional Review Board. The patients/participants provided their written informed consent to participate in this study.

## Author contributions

All authors listed have made a substantial, direct, and intellectual contribution to the work and approved it for publication.
