# Supplementary material for: Contribution of S100A4-expressing fibroblasts to anti-SSA/Ro-associated atrioventricular nodal calcification and soluble S100A4 as a biomarker of clinical severity
Source: Front Immunol. 2023 Apr 6;14:1114808. doi: 10.3389/fimmu.2023.1114808 (PMC10117984; doi:10.3389/fimmu.2023.1114808)
Supplement: Supplementary file 1 [file DataSheet_1.pdf]

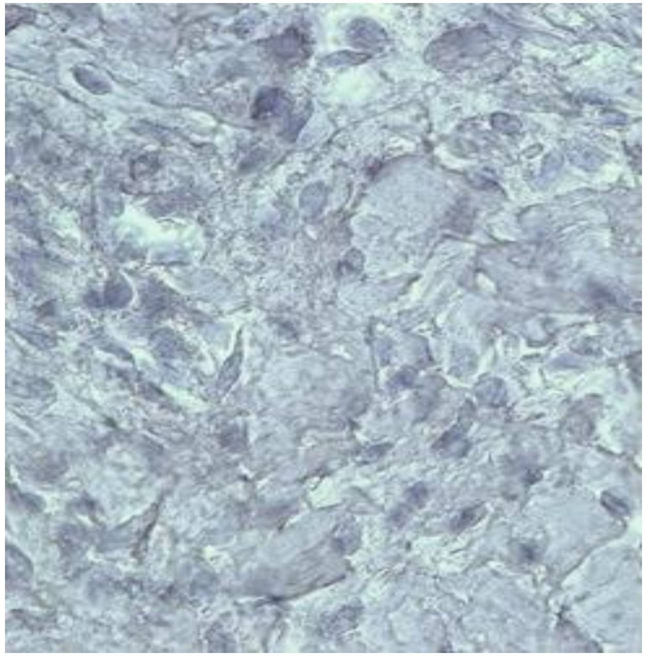

SUPPLEMENTARY FIGURE 1
A region of AV nodal tissue taken from CHB Heart #2 was incubated with anti-rabbit IgG antibody as an isotype control, confirming S100A4 primary antibody specificity.

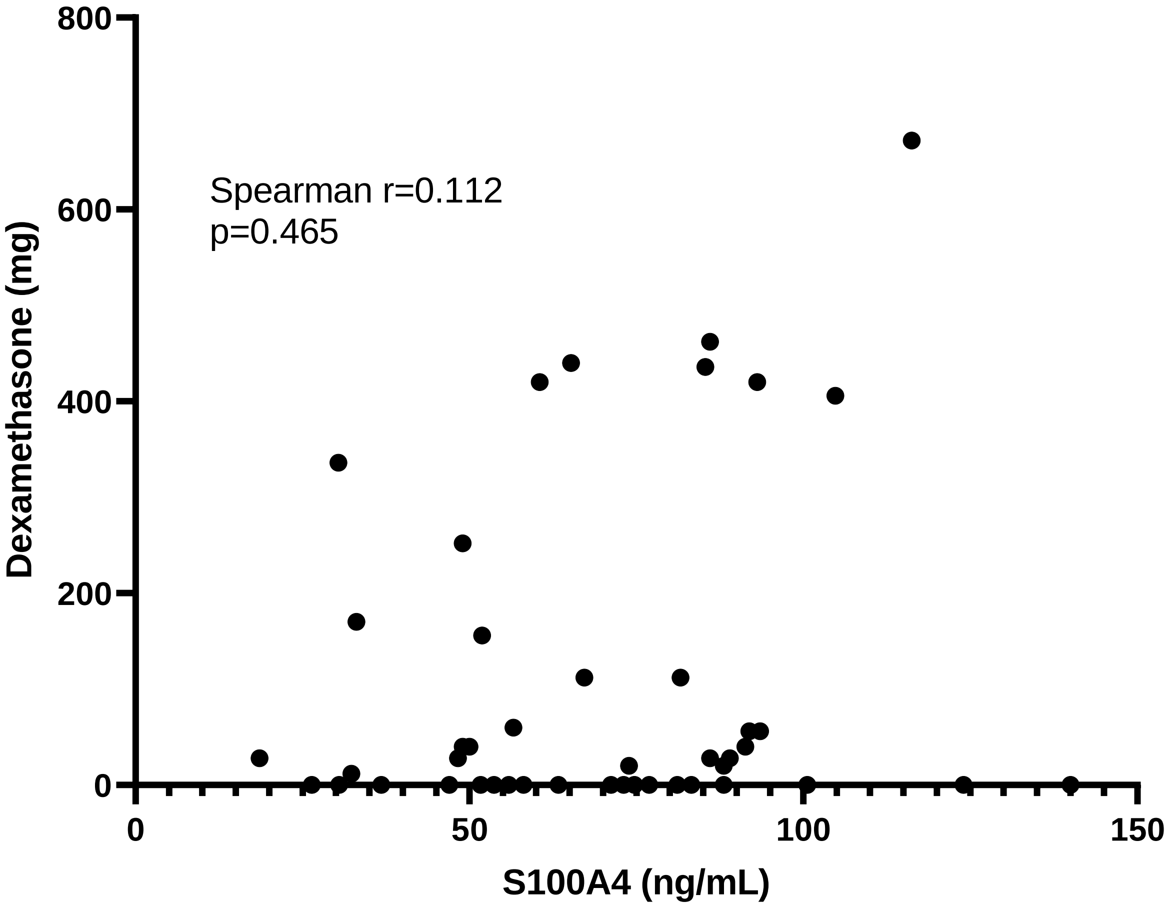

SUPPLEMENTARY FIGURE 2

No correlation was found between cumulative maternal dexamethasone dose (mg) and S100A4 (ng/mL) in cord blood (Spearman coefficient r=0.112, p=0.465). S100A4 levels and dexamethasone dose was evaluated in 27 of 31 treated neonates in which details regarding dose and timing were available and 18 not exposed to dexamethasone.

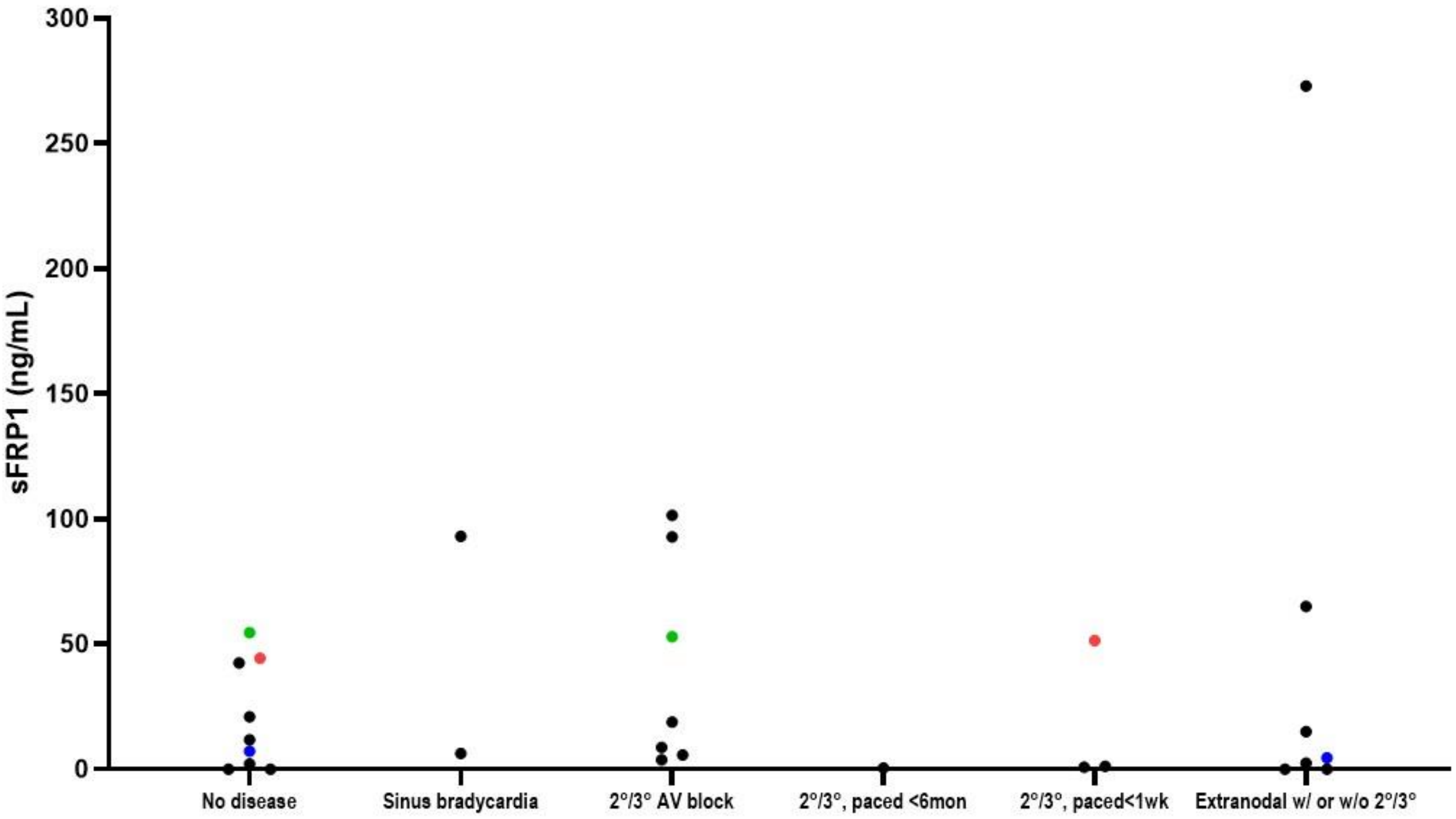

SUPPLEMENTARY FIGURE 3
Evaluation of another secreted protein, sFRP-1, was performed on a selection of the same cohort of neonates evaluated in Figure 7, with the expectation that it would negatively track with severity. As shown, the levels of sFRP-1 did not track with severity, providing a negative control. No difference in sFRP-1 levels was observed among the twin pairs.

A

Gene Symbol	Gene Name	Fold increase in CHB leukocytes
BMP1	Bone Morphogenetic Protein 1	10
BMP2	Bone Morphogenetic Protein 2	7
BMP4	Bone Morphogenetic Protein 4	16
BMP6	Bone Morphogenetic Protein 6	8
BMPR1A	Bone Morphogenetic Protein Receptor Type 1A	11
BMPR2	Bone Morphogenetic Protein Receptor Type 2	2

B

<u>GO Category / p-value</u>

Extracellular matrix / 3.91×10^{-25}

Collagen catabolic process / $9.81 \times 10^{--9}$

Metalloendopeptidase activity / 1.18×10^{-4}

Positive regulation of osteoblast differentiation / 2.81×10^{-4}

Cellular response to BMP stimulus / 0.00363

BMP signaling pathway / 0.0057

SUPPLEMENTARY FIGURE 4

Bulk RNA sequencing on leukocytes flow-sorted from CHB hearts showed increased expression of BMP signaling transcripts (A) relative to leukocytes flow-sorted from healthy, age-matched hearts. An agnostic bioinformatics inspection (B) of the top 500 transcripts ranked by fold change of expression in CHB leukocytes relative to healthy leukocytes revealed the enrichment of five gene ontology (GO) categories of relevance including extracellular matrix, collagen catabolic process, metalloendopeptidase (MMP) activity, cellular response to BMP stimulus and BMP signaling pathway.

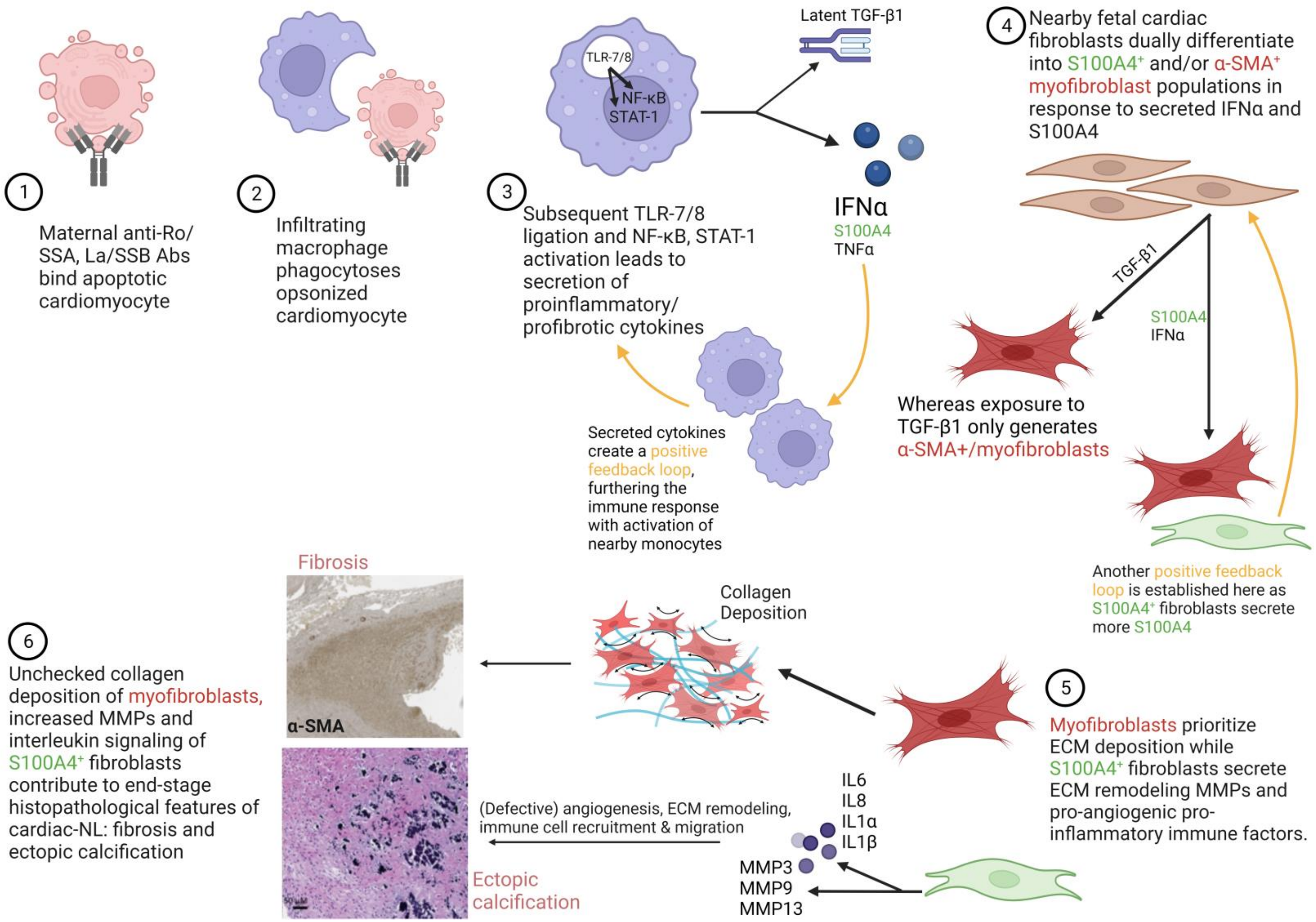

SUPPLEMENTARY FIGURE 5

Schematic model in support of a role for α-SMA⁺ fibroblasts and S100A4⁺ fibroblasts to contribute to pathological events. The former release factors related to the content of the cell matrix during fibrosis and the latter multivalent cytokine secretion, leading to unchecked collagen deposition of myofibroblasts (with resultant fibrosis) and the increased MMP and interleukin activity of S100A4⁺ fibroblasts. We hypothesize what follows includes angiogenesis, ECM remodeling, and immune cell recruitment/migration. Defective angiogenesis via IL6 represents one pathway through which the end-stage feature of ectopic calcification may arise.
